# Global community of practice: A means for capacity and community strengthening for health professionals in low- and middle-income countries

**DOI:** 10.7189/jogh.12.04034

**Published:** 2022-05-14

**Authors:** Allison Silverstein, Amy Benson, Catriona Gates, Diane Nguyen

**Affiliations:** 1 Baylor College of Medicine International Pediatric AIDS Initiative at Texas Children’s Hospital, Houston, Texas, USA; 2 Department of Hospice and Palliative Medicine, University of Tennessee Health Science Center, Memphis, Tennessee, USA; 3 Department of Pediatrics, Baylor College of Medicine, Houston, Texas, USA; 4 Department of Education, Innovation, and Technology, Baylor College of Medicine, Houston, Texas, USA

## Abstract

**Background:**

Low- and middle-income countries face distinct challenges in providing health care services and training. The community of practice (CoP) has been described as a method of facilitating much-needed connections and conversations on this topic and has been adapted over time to include virtual CoPs. We describe the development and evaluation of a global Clinical Lead Forum (CLF) using a CoP framework to structure informal continuing professional development (CPD) and enhance the capacity of health care professionals in low- and middle-income countries.

**Methods:**

Baylor College of Medicine International Pediatric AIDS Initiative (BIPAI) and its network of affiliated, independent non-governmental organizations (NGOs) provide paediatric and maternal health care for vulnerable populations around the world. We established virtual sessions across the network to discuss clinical topics, which evolved based on the need to include a COVID-19 series. We collected demographic, participation, participant and facilitator assessments, as well as leadership notes from each session as part of an educational quality improvement study. We developed and evaluated the program using the Logic Model and used the Kirkpatrick Model to assess learning outcomes.

**Results:**

A total of 299 unique participants engaged in sessions, representing a total of 10 disciplines. There were a total of 1295 participants who joined for the 11 sessions in the regular CLF series and the 23 sessions in the COVID-19 series. Survey responses were overall consistent with a value-added intervention.

**Conclusions:**

The CLF, via both the regular sessions and the COVID-19 series, served as an impactful global health CoP for CPD. By focusing on creating a safe and inviting space, ensuring equity and inclusion, activating champions, fostering engagement, and promoting innovation and adaptability, this program decreased professional isolation, strengthened peer relationships, and enhanced the knowledge and practices of health care professionals. Our model may be scaled to other systems across the world to bridge divides and create similarly meaningful communities.

Many low- and middle-income countries face distinct challenges in health care services and training, including the burden of infectious and non-communicable diseases, shortages of health care professionals, and limitations in workforce capacity [1-3]. This creates a critical need for enhancing the capacity of existing health care professionals. Informal continuation of professional development (CPD) through interactions with peers can provide opportunities for growth, and programs have demonstrated that the use of information and communication technologies for linking health care professionals in disparate regions can improve clinical practice and learning [4,5]. Diverse groups of health care professionals in sub-Saharan Africa and other low- and middle-income countries have readily welcomed online CPD opportunities as feasible and valuable [6,7]. While each country and setting are unique, underlying commonalities do exist, and much can be learned from a cross-border exchange of experiences.

The community of practice (CoP) has been widely explored as a means of facilitating such connections and conversations in many industries, including health care [8-12]. A CoP, defined as a group of people with a shared concern, committed to deepening their knowledge and expertise by interacting on an ongoing basis, has been adapted over time and can also take the form of a virtual CoP, where the online community connects geographically dispersed members [9,13]. CoPs have been found to be effective in teaching and disseminating knowledge, promoting collaborations, creating innovative solutions, strengthening the sense of community within an organization, fostering individual professional identity, and overcoming professional isolation, especially in communities located in rural areas [9,11,14-19].

We describe the development and evaluation of a global Clinical Lead Forum (CLF) using a CoP framework to structure informal CPD and enhance the capacity of health care professionals in low- and middle-income countries. We aimed to create a community of professionals from all clinical disciplines, regardless of their speciality, in which participants learn from each other, ameliorate feelings of professional isolation, strengthen peer relationships and collaboration, enhance clinical knowledge and best practices, and explore practice management challenges.

## METHODS

### Program setting and participants

The Baylor College of Medicine International Pediatric AIDS Initiative (BIPAI) network is a global network of independent non-governmental organizations (NGOs) providing paediatric and maternal health care to vulnerable populations worldwide. The NGOs operate outpatient clinics and partner with Ministry of Health facilities to serve over 365 000 people across Argentina, Botswana, Colombia, Eswatini, Lesotho, Malawi, Romania, Tanzania, and Uganda. Over 2400 locally employed professionals support clinical and programmatic services. BIPAI headquarters, based at Baylor College of Medicine and Texas Children’s Hospital in Houston, Texas, USA supports the NGOs through education and capacity enhancing initiatives.

Program champions were identified at each NGO, and these Clinical Leads collaborated with CLF leadership based at BIPAI headquarters to ensure programming was relevant to the local context. Clinical Leads helped mobilize participants from NGOs who had a particular interest in and relevance to the topic. Healthcare professionals across the BIPAI network and at affiliate Ministry of Health sites were invited to participate in the CLF; there were no specific exclusion criteria.

### Patient and public involvement

Patients or the public were not involved in the design and conduct of this program and evaluation.

### Program structure

Virtual monthly sessions discussing a clinical topic were conducted for approximately one hour using Zoom. Clinical Leads recruited health care professionals from their site to facilitate. Facilitators had demonstrated knowledge, experience, or leadership related to the topic, and the facilitator role rotated between NGOs. Facilitators presented at the start of a session, establishing a foundation of knowledge before encouraging a discussion among participants by having them share their personal experiences, successes, and challenges. The presentation topics were developed with Clinical Lead and participant input and varied from the description of clinical strength, best practice, lesson learned, challenge overcome, or case study (see Appendix S1 in the **Online Supplementary Document**). The discussion was framed as the core component of the CLF, whose richness depended on the exchange among participants. Dialogue enhanced the opportunities to break down country silos, strategize for the adoption of best practices, and develop new approaches. Each meeting was recorded and archived with the corresponding presentation and summary notes in an online knowledge resource library.

As facilitators prepared for the session, CLF leadership coached them by providing resources (see Appendix S2 and S3 in the **Online Supplementary Document**) and written feedback on the presentation material. In addition to meeting before the session, the facilitators were also debriefed after the session to provide feedback.

### Adapting during the COVID-19 Pandemic

With the emergence of the COVID-19 pandemic in March 2020 and based on feedback from NGO leadership to address escalating NGO needs, CLF leadership shifted from monthly to weekly CLF sessions to explore scientific and clinical COVID-19 data. These sessions were designated as the COVID-19 Series. Multidisciplinary team members from BIPAI headquarters and invited content experts facilitated these sessions to offset the responsibility of facilitation from overly burdened frontline health care professionals at the NGOs. The knowledge resource library was continually curated to include relevant COVID-19 resources such as clinical protocols, national government policies, peer-reviewed literature, templates, and tools. As the number of new cases in the first wave of COVID-19 began to plateau and NGOs developed individualized management systems, the CLF transitioned back to its regular schedule. During the second wave of COVID-19 cases, and after a discussion with NGO leaders indicated the need for further COVID-19 information, a second biweekly COVID-19 series (Series #2) was initiated.

### Program evaluation and data analysis

We collected demographic, participation, participant, and facilitator assessments, as well as leadership notes from each session as part of an educational quality improvement study. Using the Kirkpatrick Model for assessing learning outcomes, we assessed 1) participant satisfaction and reaction, 2) facilitator reactions to the mentoring and facilitating process, and 3) participant and facilitator attitudes and knowledge gained [20]. Surveys were distributed at the end of each session and at the end of a series using SurveyMonkey (Momentive, San Mateo California, USA); results were anonymous to protect privacy and confidentiality.

We developed and evaluated the program using the Logic Model (**Table 1**) which outlines the relationships between resources, activities, outputs, outcomes, and impacts of the program [20]. This approach helped us develop the program, evaluate its activities and intended effects, and remain highly adaptive to changing learning environments for optimized engagement.

**Table 1:** Logic model for clinical lead forum

Inputs	Activities	Outputs	Outcomes
**Input for activities remain constant:** • **Funding: **none • **Educator commitment: **Time + effort of faculty, resident, HQ staff, NGO Clinical Lead, local staff • **Educational technology: **Zoom • **Institutional commitment: **HQ and NGO leadership support	**Needs assessment** • Discussions with NGO leadership, Clinical Leads, and participants of past CPD programming	• Documentation of informal discussions • Support from institutional leaders	• Understand different CPD needs • Understand site resources
	**Program development** • Outline program goals and objectives, program format, and curriculum • Establish processes to create enduring materials as well as materials for speakers • Establish a network of contacts, resources, and experts • Invite facilitators	• Established curriculum • Creation of a library of references • Finalization of the list of facilitators and interested participants	• Enhance clinical knowledge, skills, and attitudes • Provide relevant CPD opportunities • Provide an opportunity to create a community and decrease isolation
	**Program implementation** • Introduce the program • Meet with facilitators prior to sessions Clinical Leads mobilized local staff	• Session attendance + assessment • Adaptable program based on changes in organizational needs and circumstances (eg, new drugs, studies, clinical data, pandemic)	
	**Pivot to COVID-19 Series based on emerging organizational needs** • Discussions with NGO leadership identified the need to shift focus of CLF • Monitor the COVID-19 situation and number of cases at NGO sites as one correlation to interest in COVID-19 topics • **Initiate Series #2 with second wave of COVID-19 cases**	• Session attendance + assessment • Synthesized high-volume and rapidly-evolving clinical information for data-driven, scientific updates to advise clinical care management	
	**Introduce live, simultaneous Spanish language interpretation after evaluation of attendance and participation**	• Attendance of Spanish-speaking participants • Impact of interpretation on participation	• 3 outcomes above for Spanish-speaking participants • Promote inclusivity and break down language barrier
	**Facilitator coaching** • Peer coaching for facilitator preparing session • Facilitated discussion and exchange on best practice • Post-session debrief and feedback for facilitator	• Effectiveness of teaching using Kirkpatrick’s model • Facilitator(s) assessment of peer coaching	• Enhance professional development, presentation, and facilitation skills

HQ – BIPAI headquarters

Data analysis involved standard descriptive statistics, looking at participation and quantitative survey responses. The initial CLF series (session 1-2) and COVID-19 series #1 and #2 were evaluated on a scale with the following variables: yes, no, unsure, N/A. The follow-up CLF series (sessions 4-11) was evaluated using a 5-item Likert scale. Qualitative survey responses to open-ended questions were grouped by themes. Survey questions were added or modified throughout the CLF to evaluate evolving programming (see Appendix S4 in the **Online Supplementary Document**).

All data was kept on a secure, shared institutional cloud server and communication between co-investigators was sent via secure encrypted email. Program participation was voluntary and did not impact performance evaluations or employment. Ethics approval was obtained from the Baylor College of Medicine Institutional Review Board, protocol H-47734.

## RESULTS

In total, there were 299 unique participants and 45 unique facilitators across all sessions; they practised at NGOs or the affiliated Ministry of Health facilities in the nine countries of the Network. They represented a total of 10 disciplines: administration (eg, program managers and implementors), community health workers, laboratory, monitoring and evaluation, medicine, nursing, nutrition, pharmacy, psychosocial, and social work. Sessions received a median of 32.5 attendees (range = 18-67, IQR = 28-40.75). The median participant attended one session (range = 1-32, IQR = 1-3.5); 147 participants attended more than one session.

A total of 389 participants and 27 facilitators attended the 11 sessions included in the regular CLF series. A total of 906 participants (n = 631; n = 275) and 43 facilitators (n = 21; n = 22) attended the two clinical COVID-19 series, accounting for 23 sessions (n = 17 in the first series, n = 6 in the second).

The regular CLF had a total of 122 participant survey responses and 10 facilitator survey responses for the individual sessions. The two COVID-19 series had a total of 344 individual session survey responses (n = 249; n = 95) and 46 overall session surveys responses (n = 30; n = 16). **Table 2** and **Table 3** show quantitative survey responses from the regular CLF Series and COVID-19 series. **Table 4** explores themes among qualitative responses to open-ended questions in the surveys across all session surveys. Additional qualitative and quantitative data are summarized in the following paragraphs.

**Table 2:** Regular Clinical Lead Forum series session participant survey responses

	Sessions 1-2 (n = 18)*			Sessions 4-11 (n = 104)
	**yes (%)**	**no**	**unsure**	**1-5 (IQR)†**
Did this presentation and facilitated discussion emphasize new concepts?	10 (56%)	6 (33%)	2 (11%)	5 (4-5)
Did this presentation and facilitated discussion refresh previous knowledge?	15 (83%)	3 (17%)	0 (0%)	5 (4-5)
Do you intend to make any clinical practice change(s) as a result of information learned during this session?	8 (44%)	7 (39%)	3 (17%)	4 (4-5)
Did the facilitated discussion adequately address the limitations and opportunities inherent to the local health system?	15 (83%)	2 (11%)	1 (6%)	5 (4-5)
Was the reporting of scientific research presented objectively?	6 (35%)	2 (12%)	9 (53%)	5 (4-5)
Did the facilitator adequately facilitate discussion between and amongst participants?	18 (100%)	0 (0%)	0 (0%)	5 (4-5)
Attending the Clinical Lead Forum helped me feel an increased sense of community.				5 (4-5)
Please assign an overall score of the presentation (1-10):	7 (6.25-8)			
Please assign an overall score of the facilitated discussion (1-10):	8 (7-8)			
Please assign an overall score of the session:				5 (4-5)

IQR – interquartile range

*Session 3 was omitted from the evaluation of the CLF because it was designed for participants to showcase abstracts accepted to an external conference.

†5-item Likert scale: 1 – strongly disagree, 2 – disagree, 3 – neither disagree nor agree, 4 – agree, 5 – strongly agree

**Table 3:** Clinical Lead Forum COVID-19 series session participant survey responses

	Sessions 1-23 (n = 344)			
	**yes (%)**	**no**	**unsure**	**n/a**
Did this presentation and facilitated discussion emphasize new concepts?	303 (88.1%)	28 (8.1%)	13 (3.8%)	0 (0%)
Did this presentation and facilitated discussion refresh previous knowledge?	338 (98.3%)	3 (0.9%)	3 (0.9%)	0 (0%)
Do you intend to make any clinical practice change(s) as a result of information learned during this session?	184 (53.5%)	24 (7.0%)	76 (22.1%)	60 (17.4%)
Did the facilitated discussion adequately address the limitations and opportunities inherent to the local health system?	297 (86.3%)	19 (5.5%)	28 (8.1%)	0 (0%)
Was the reporting of scientific research presented objectively? (n = 249)	220 (88.4%)	3 (1.2%)	26 (10.4%)	0 (0%)
Please assign an overall score of the presentation and facilitated discussion (1-10):	9 (8-10)			

n/a – not applicable

**Table 4:** Themes of qualitative response for all session surveys

Series	Themes	Quotes
Clinical Lead Forum Participants	Open discussion	“It was a great discussion that really made me think of what [the clinic] can do to help our needy clients during this challenging time.”
		“Great discussions, appreciated the relaxed atmosphere and encouragement for people to participate.”
		“I loved the open discussion format.”
		“…had great sharing between sites.”
	Applicable to resource-constrained settings	“This is a great topic as it is really what we face on daily basis.”
		“Was really practical about what we could do now.”
		“This presentation really helped for our facility as due to limited resources we have been unable to procure full PPE, but the discussions gave us alternatives to deal with the situation.”
		“Great presentation, applicable to resource-constrained settings.”
	Learning from others	“This type of conversation gives us ideas to improve our clinical practice.”
		“It was really great to hear how other facilities are tackling the challenges.”
		“This was the best! So cool to hear what everyone is doing! Lots of innovation during this difficult time. Seriously - this should be happening more often to share ideas.”
		“It will really help us learn a lot from practical experience of other [clinics].”
	Building community	“I feel the CLF does a better job of building a sense of community than I get at [in-person] network meetings and look forward to them continuing!”
		“Great interaction across the network-learning hub indeed...”
		“I liked that all participants introduced themselves and the warm greetings, made the session much more enjoyable.”
	Capacity building	“We are really being capacitated as we often get so busy we don’t get a chance to review all the developments regarding this pandemic.”
		“This presentation helped iron out some of the myths that are around these vaccines.”
		“Always good to learn the most up to date information on COVID-19 as it changes so frequently.”
	Interpretation	“The inclusion of the translator was huge and inclusive.”
		“Many thanks to the organizers for having the simultaneous translation into Spanish. It brings us closer and allows us greater participation.”
		“It was very useful to have the translation to take advantage of the meeting.”
Facilitators	Mentorship	“Great mentorship support.”
		“I received excellent mentorship and support!”
		“The organizers did their part in guiding us accordingly during the preparations for the presentation. We really appreciate.”

### Regular series

#### Participants

Participants enjoyed the opportunity to hear from other NGOs – how they solve similar challenges and share their best practices. The participants valued the “interactive sessions” and desired more discussion around “challenges, quality improvement, and problem-solving.”

#### Facilitators

Facilitators reported learning something from the discussion about their presentation (median = 5, IQR = 3.25-5), developing greater confidence about presenting and facilitating a virtual discussion (median = 5, IQR = 2.25-5), and feeling an increased sense of community (median = 5, IQR = 3.25-5). Areas of learning included suggestions for optimizing their presentation, such as framing the content within session parameters and formatting using their chosen media, using different learning tools (eg, polls, games), outlining good practices in presenting and facilitating, and suggesting improvements. They agreed that presenting and facilitating the discussion was valuable for their professional development (median = 5, IQR = 3.25-5). Participants expressed that they received “great mentorship support” and received “excellent guidance and feedback.” They reported that working with a second facilitator to prepare and facilitate collaboration helped “boost their morale and confidence.”

### COVID-19 series

Participants expressed that they applied the knowledge learned, both personally and professionally, and shared the resources with colleagues, family, and friends. They reported that the sessions helped improve infection prevention and control measures, guided programming at the NGO, and provided clarity on patient management. The participants identified the forum as a reliable “source of facts amidst so much media misinformation”. Several new initiatives were created as a result of the series, including psychosocial support programs for staff both at the headquarters and NGO levels. Respondents agreed that attending the COVID-19 series decreased their stress and worry surrounding COVID-19 (median = 4, IQR = 3-4) and helped them stay informed about new developments related to COVID-19 (median = 5, IQR = 5-5). The participants also found it easy to access the Zoom webinar with the internet and technology available (median = 5, IQR = 5-5).

### Language interpretation services

Since the introduction of simultaneous Spanish interpretation, Spanish-speaking participants have described it as helpful in supporting their understanding of the content and their interaction with other participants during the session, while also observing that it allowed for greater participation.

## DISCUSSION

We effectively created a CoP across a vast global network by cultivating a focused community among multidisciplinary health care professionals. In doing so, our team worked to diminish feelings of professional isolation, strengthen peer relationships and collaboration, and enhance clinical knowledge and best practices through sustained engagement. The CoP framework was useful in successfully developing the CLF and creating value for participants and the community itself.

Survey responses highlighted the effectiveness of our intervention to objectively share new concepts and facilitate discussion while recognizing limitations and opportunities inherent to the local health systems. In doing so, the CLF effectively fostered a community and led to both participant and facilitator growth.

As a capacity-enhancing initiative, we focused on both group and individual growth within the CLF. From the group perspective, as individuals consistently came together over time to share their practical experience (including clinical cases), approaches to care or its programming in various conditions, perspectives, successes, and challenges, a community developed with its own identity and collective knowledge in treating patients. This is similar to previously reported benefits of CoPs with a targeted focus on evidence-based practice exchange [21,22]. Individuals also expanded their network, increasing their ability to connect with others who could provide guidance or solutions. Furthermore, during the original CLF programming (not the COVID-19 series), individuals who volunteered to lead a meeting as facilitators expressed an increase in self-confidence and morale. The CLF provided a safe, internal space with peer-coaching support with the aim of promoting professional development in preparing written material, presenting, and facilitating discussions. Individuals enhanced their capacity to effectively use these skills in their primary work and external settings (eg, conferences). Focusing on the learning of both the group and the individual fostered the social learning process that underlies a CoP [23].

Building a CoP for knowledge sharing is a socialization process that can be challenging to operationalize, especially without the ability to be in the same physical space [24]. We have cultivated a CoP despite most CLF participants having never met each other in person, which helped reduce the sense of isolation. This is similar to benefits reported in CoPs used as a strategy for online knowledge transfer [22,25]. Our experience aligns with that of other researchers, showing that the effective fostering of social learning is a process requiring some intentional design without losing sight of the spontaneity of organic developments [24,26]. We describe some of the strategies we employed and the characteristics of the CoP design below.

### Creating a safe and inviting space

We fostered an environment of psychological safety so that participants felt at ease to be vulnerable, to share, and to learn [16]. We established CLF norms at the onset that were continually referenced, including defining the purpose of the CoP, inviting dialogue and exchange, reminding that the richness of the CLF depends on active participation, and reassuring that no comment or question is too small. We encouraged intellectual honesty and a commitment to truthfulness when sharing ideas that inform others, and frequently modelled this value. For example, we normalized not knowing the answer and acknowledging awareness of one’s limitations. We focused on establishing social presence and projecting one’s personal characteristics into the virtual space to foster connections, particularly by facilitators [27]. We encouraged participants to use multiple functions of the Zoom technology (eg, chat, raising hands, emojis, audio, camera) to contribute to the community connection and achieve this social presence. Given that many participants joined from low-resource settings where internet connectivity may be limited, we were careful to strike a balance between encouraging social presence by using a camera and not alienating those who could not turn it on due to bandwidth limitations. To create a warm and inviting space, we embedded fun activities during sessions to energize participants, put them at ease, and spark connection through moments of levity and low-risk participation. We appreciated all participants who could join through whatever means available and reassured them that there were many ways to participate.

### Ensuring equity and inclusion

The dynamic between participants is critical to the development of the CoP [28]. The baseline dynamic of the CLF was challenging since it included a heterogeneous group of individuals who come from different cultural, education, and training backgrounds. Though they were all connected to the BIPAI network, this network identity was loose, and they had a stronger sense of belonging to their individual NGOs. Compounding this baseline dynamic was the global health backdrop, which both historically and presently fosters unequal power dynamics between individuals from high-, middle-, or low-income countries [29-31]. We aimed to break down hierarchical barriers of discipline (eg, physician vs allied health professionals) and positions (eg, clinical director vs staff). We continually emphasized, explicitly and implicitly, our CoP norm that everyone has a proverbial seat at the table and the opportunity to share their knowledge, experiences, and questions regardless of which country they worked in, their training background, or titles. Implicit ways included embedding topics on which individuals of certain countries or disciplines could confidently share. The use of the “participant” and “facilitator” terminology was specifically adopted to reflect individuals’ roles in each session in a more equitable way and underline the active contributions from all in the learning process. The facilitator role continually rotated, enabling experts to express their voices across all participating countries.

### Activating champions and promoting engagement

Identifying champions at each of the participating NGOs who were committed to the learning partnership helped increase awareness of the CLF and disseminate communication to their local colleagues. These Clinical Leads helped identify key topics of collective interest for the CoP and organized individuals at their site to facilitate sessions. The Clinical Leads could often be relied on for their consistent attendance, which provided a presence that bonded the community and helped participants come to know each other as individuals. During the sessions, the Clinical Leads actively contributed to the discussion and served as a catalyst for engagement. They knew their clinical practice well and encouraged sharing from the more reticent colleagues participating, who also have relevant experience and knowledge. It has been noted that when participation is not mandated, participant engagement can be fragmented, and that CoPs succeed when there is an authentic thirst to share knowledge and experiences [28]. The Clinical Leads were instrumental in the success and growth of the CLF by inspiring voluntary participants to exchange and refine their shared practice.

### Innovation and adaptability

Shortly after we launched the CLF, the justification and value for this CoP were reinforced by urgent needs stemming from the COVID-19 pandemic. During the early days of the pandemic, fraught with extreme volatility and uncertainty, NGO leadership and staff expressed the desire and need for a space where clinical information related to COVID-19 could be shared, critically evaluated, and discussed. Vast amounts of information and misinformation were being published in scientific and mainstream sources which made it challenging to stay updated. The nature of preliminary data and quickly changing recommendations from trusted scientific sources required ongoing nuanced interpretation and application to one’s own setting. The existence of the CLF enabled a quick response, pivoting from a monthly CLF meeting to a weekly COVID-19 series that shared the latest data available, identified the unknowns, and interpreted the nuances to help participants apply what was known to their practice with considerations of limitations and risk-benefits analyses. CLF participants hailed from regions of the world that experienced the first wave at different times; this allowed those in Latin America, who were managing COVID-19 cases long before the first cases in sub-Saharan Africa, to share their experiences with African participants. The consistency of weekly meetings within this community served to reduce a sense of anxiety and social isolation.

Despite many successes, our team faced challenges and limitations in designing and implementing the programme. When measuring participation and attendance, we noticed that a few participants were joining from the network’s Spanish-speaking countries and that the language barrier was recognized as a significant limitation. Initially, we pilot-tested artificial intelligence translation services, which were ineffective at translating diverse English accents. After evaluation and switching to a live, simultaneous interpreter, the community became familiar with the expectations, and we were able to break down the language barrier. This allowed participants from Latin America to more fully engage or join for the first time. Additionally, we created a library containing resources and content that emerged from the CLF, but it has not been widely used. Moving forward, we plan to explore what participants want and need so that we could create a repository of knowledge that is functional and easily shared. Establishing a mechanism for participants to connect and network in between the live meeting times so as to continue discussions and interactions would enhance the richness of the community. We recognize that participants of the CLF participated voluntarily, and survey responses were limited, which may have skewed answers towards positive feedback. Nevertheless, we are confident that responses reveal the value of this community and intend to continue growing it. Finally, many of the participants congregated around one device to join the CLF, changing the dynamics of individual participation and limiting our ability to capture all those who joined. To date, our evaluations have not investigated the long-term outcomes of the CLF, and we have not captured programmatic changes that occurred after CLF learning and discussions. Overall, such challenges have afforded us opportunities to explore room for growth – to optimize engagement and learning, we had to be highly adaptive, iterative, and creative.

## CONCLUSION

The CLF, via both the regular sessions and COVID-19 series, served as a valuable and empowering global health CoP for continuing professional development. By focusing on creating a safe, inviting space, ensuring equity and inclusion, activating champions and engagement, and promoting innovation and adaptability, this program decreased professional isolation, strengthened peer relationships, and enhanced the knowledge and practices of health care professionals. Our model may be scaled to other systems across the world to bridge divides and create similarly meaningful communities.

## Additional material


Online Supplementary Document
