# Supplementary material for: Global community of practice: A means for capacity and community strengthening for health professionals in low- and middle-income countries
Source: J Glob Health. 2022 May 14;12:04034. doi: 10.7189/jogh.12.04034 (PMC9107096; doi:10.7189/jogh.12.04034)
Supplement: Online Supplementary Document [file jogh-12-04034-s001.pdf]

Appendix 1. CLF Schedule of Topics 2020-21

BIPAI Clinical Forum 2020-21: Regular Series & COVID-19 Series

CLF Regular Series- denoted in black
COVID-19 Series #1- denoted in blue
COVID-19 Series #2- denoted in red

Regular Session No.	Date	Topic	Facilitators*
**2020**			
1	Feb 7	Resistance and Genotyping	Medical Officer, Pediatrician, Pediatric Resident (Malawi)
2	March 6	Malnutrition and HIV	Medical Officer, Clinical Director (Tanzania)
	March 27	COVID-19 Overview	Pediatrician (USA)
	April 3	Infection Control	Pediatrician (Malawi†)
	April 17	Self-care and Coping in the Healthcare Provider	Psychologist (USA)
	April 24	COVID-19 Novel Therapies	Pediatrician, Pediatric Infectious Disease Specialist (Malawi†)
	May 8	COVID-19 and HIV	Pediatrician (USA)
	May 15	COVID-19 Diagnostics	Pediatrician (Uganda†)
	May 22	Acute COVID-19 Management	Pediatrician (Eswatini†)
	May 29	COVID-19: Pediatric Considerations in the Resource Constrained Setting	Pediatrician (Tanzania†)
	June 5	COVID-19 and Tuberculosis	Pediatrician (Eswatini†)
	June 12	Addressing COVID-19 Risks to Young People	Pediatrician Scientist- Adolescent Specialist (Malawi†)
	June 19	COVID-19 Pathophysiology	Pediatrician (Tanzania†)
	June 26	COVID-19 and Pregnancy	Maternal Fetal Medicine Specialist (Malawi/USA)
	July 17	Vaccine Development and LMIC Implications	Scientist (USA)
3	July 24	AIDS 2020 Poster Presentations	Panel of Network staff who presented at the AIDS 2020 Conference
	July 31	COVID-19 and Dermatologic Manifestations	2 Pediatricians (Eswatini†)
	August 7	COVID-19 and Nutrition	Pediatrician (Uganda†)

	August 14	COVID-19: Novel Therapies- an update	Pediatrician, Pediatric Infectious Disease Specialist (Malawi†)
	August 21	Ethical Considerations in the Era of COVID-19	2 Pediatricians (USA)
4	Sept 18	HIV-Exposure Infant Prophylaxis, Testing and Feeding Practices	Clinical Director, Pediatrician (Lesotho)
5	Oct 16	Sexual & Reproductive Health & Family Planning Programs	Pediatrician (Argentina)
6	Nov 20	HPV and Cervical Cancer Screening	Pediatrician (Eswatini)
7	Dec 11	Emergency Delivery of Nutritional Services in a Resource-Limited Setting	Nutritionist, Pediatrician, and Clinical Director (Uganda)
**2021**			
8	Jan 22	The First 95: Finding and Linking PLHIV to Life-Saving ART	Medical Officer, 2 Pediatricians (Malawi)
	Feb 5	COVID-19 Best Practices Roundtable	Panel of clinicians across the Network
	Feb 12	Oxygen Administration and Monitoring in Low Resource Environments	2 Pediatric residents, Pediatrician (USA)
	Feb 26	Thought Management as a Strategy for Wellness	Psychologist (USA)
	Mar 5	COVID-19 PPE Re-use	2 Clinical Directors, Pediatrician (Eswatini, Uganda, Botswana)
	Mar 12	COVID-19 Vaccine and Variants	Scientist (USA)
9	Mar 26	Romania Case Study: Child-Adolescent-Adult Health Care Transition	Monitoring & evaluation specialist (Romania)
	April 9	COVID-19 Updates	Pediatric resident, Pediatrician, Rural Medicine Physician, Nutritionist (USA, Eswatini†, Argentina, Colombia)
10	Apr 23	Differentiated Service Delivery Models: A Focus on Differentiated HIV Care and Treatment Services	Clinical Director (Uganda)
11	May 28	Pediatric and Adolescent Palliative Care- Mbeya Center of Excellence	Psychosocial, Social Worker, Medical Officer, Pediatrician, Clinical Director (Tanzania)

**Facilitator names have been removed and replaced with their discipline and country of practice to represent the diversity of expertise showcased in the CLF.*

†These facilitators practice in countries indicated but temporarily relocated elsewhere during the COVID-19 pandemic.

Appendix 3. Effective Facilitation Guide

Guide for Facilitation

BIPAI Network Clinical Lead Forum

Effective facilitation of a discussion involves the recognition and employment of different perspectives and different skills to create an inclusive environment. In order to do so, it is important to consider the features of effective discussions, and conditions that promote small group interaction and engagement. Discussion is a powerful mechanism for active learning; a well-facilitated discussion allows the participant to explore new ideas while recognizing and valuing the contributions of others.

<u>Roles as Discussion Leader</u>

1. **Create an inclusive environment**

   Opportunities for reflection

   - What do the participants bring to the group? ("Characteristics that may give you a unique perspective")
   - Self-awareness; awareness of others
   - What do I bring to the group? What surprises or challenges me?
   - What behaviors am I most familiar or comfortable with?
   - What behaviors challenge me?

   Dos

   - Allow participants to introduce themselves – you can even set up an ice breaker to have pairs of students introduce each other.
   - Be clear up front about expectations and intentions amongst participants and the facilitator.
   - Use inclusive language.
   - Ask for clarification if unclear about a participant's intent or question.
   - Treat participants with respect and consideration.
   - Develop an awareness for barriers for learning (cultural; social; experiential, etc).
   - Provide sufficient time and space for participants to gather their thoughts and contribute to discussions.
   - Provide opportunities for participants to pair-share.

   Don'ts

   - Use certain conventions or language that will exclude certain groups from understanding the context of the discussion or make them feel uncomfortable.
   - Assume participants all have the same expectations when the group first convenes.
   - Over-generalize behavior or have stereotypical expectations of participants (tokenism).
   - Use (or allow others to use) disrespectful language or tone, or disrespectful non-verbal communication.
   - Convey a sense of self-importance or superiority.
   - Allow only the dominant or more verbal participants to take over the conversation.
   - Discourage alternate views or counter-arguments.
   - Try to be someone else - be yourself.

2. **Keep discussion constructive and positive**
   - Try to keep the group on task without rushing them.
   - If the group starts to veer in the direction of negativity and/or pointless venting, ask them how they would like to address this.

- Step back when a group is functional/functioning – help participants become independent learners; take control of their learning.

3. **Encourage participants**
   Encouraging participation can be accomplished by:

- Asking follow-up questions, and paraphrasing the comments for everyone to ponder. A combination of initiating and probing questions can be an effective approach to bring out participants' ideas further.
- Asking the contributor for further clarification and/or elaboration.
- Re-visiting past contributions and incorporating them into subsequent discussions.
- Encouraging others to add their reactions or ideas to build on someone's comment.
- Not being afraid to admit your own ignorance or confusion if you don't know something – invite others to provide resources, and use the opportunity to discuss with the group how one might go about researching the issue.
- Discomfort and silence are ok, but balance with a clearly stated context and purpose.

Adapted from Sheridan Center. Please visit for more resources:
https://www.brown.edu/sheridan/teaching-learning-resources/teaching-resources/classroom-practices/learning-contexts/discussions/tips

Appendix 4. CLF Participant and Facilitator Surveys

Clinical Lead Forum Participant Assessment

Regular Series: Session 1-2

Thank you for participating in the Clinical Lead Forum. Please complete this optional survey to help us evaluate the educational program. If you fill out this anonymous survey, you are agreeing to take part in this educational research activity. Your decision to participate, or not participate, in the Forum and optional surveys will have no impact on your employment status or job performance evaluations. Thank you for your feedback.

Date of Clinical Lead Forum	
Topic of Clinical Lead Forum	
Facilitator(s)	
Professional Category/Degree	MBBS/ MBChB/ MD/ DO/- in practice<br>MBBS/ MBChB/ MD/ DO/- resident/fellow<br>Pharmacist<br>PhD/PsyD/EdD/DrPH<br>Medical student<br>Nurse<br>Other
Foundation where you are based	Argentina<br>Botswana<br>Eswatini<br>Lesotho<br>Malawi<br>Romania<br>Tanzania – Mbeya<br>Tanzania - Mwanza<br>Uganda<br>BIPAI HQ<br>Other
Did this presentation and facilitated discussion emphasize new concepts?	Yes<br>No<br>Unsure
Did this presentation and facilitated discussion refresh previous knowledge?	Yes<br>No<br>Unsure
Will this presentation and facilitated discussion be useful in clinical practice?	Yes<br>No<br>Unsure<br>Not applicable – non-clinical respondent
Do you intend to make any clinical practice change(s) as a result of information learned during this session?	Yes<br>No<br>Unsure<br>Not applicable – non-clinical respondent

Did the facilitated discussion adequately address the limitations and opportunities inherent to the local health system?	Yes<br>No<br>Unsure
Did the facilitated discussion outline realistic strategies to improve clinical outcomes given the limitations and opportunities inherent to the local health system?	Yes<br>No<br>Unsure
Was the reporting of scientific research presented objectively?	Yes<br>No<br>Unsure
Did the facilitator clearly define the learning objectives?	Yes<br>No<br>Unsure
Was the presentation delivered clearly?	Yes<br>No<br>Unsure
Did the facilitator adequately facilitate discussion between and amongst participants?	Yes<br>No<br>Unsure
Please assign an overall score of the presentation: 1 (needs major improvement) to 10 (exceptional)	1 2 3 4 5 6 7 8 9 10
Please assign an overall score of the facilitated discussion: 1 (needs major improvement) to 10 (exceptional)	
Please provide additional comments and constructive feedback for the facilitators and/or organizers of the session to improve future clinical lead forums.	
Suggestions for future Clinical Lead Forum topics:	

Clinical Lead Forum Participant Assessment

Regular Series: Session 4-11

Thank you for participating in the Clinical Lead Forum. Please complete this optional survey to help us evaluate the educational program. If you fill out this anonymous survey, you are agreeing to take part in this educational research activity. Your decision to participate, or not participate, in the Forum and optional surveys will have no impact on your employment status or job performance evaluations. Thank you for your feedback.

Date of Clinical Lead Forum	
Topic of Clinical Lead Forum	
Facilitator(s)	
Discipline of your primary work area	Medicine (e.g. doctor, clinical officer, medical assistant)<br>Nursing<br>Nutrition (e.g. nutritionist)<br>Pharmacy (e.g. pharmacist, assistant)<br>Psychosocial (e.g. social worker, psychologist, counselor)<br>Student/trainee (please specify)<br>Other (please specify)
Foundation where you are based	Argentina<br>Botswana<br>Eswatini<br>Lesotho<br>Malawi<br>Romania<br>Tanzania – Mbeya<br>Tanzania - Mwanza<br>Uganda<br>BCM/TCH<br>Other
Please indicate the overall teaching effectiveness of the facilitators by indicating your agreement with the following statements. 5 being strongly agree and 1 being strongly disagree:	
Highlighted program details and lessons learned that taught me important lessons	

Adequately addressed the limitations and opportunities inherent to the local health system	
Adequately promoted interactive discussion between and amongst participants	
Demonstrated sound communication skills	
Used effective tools to achieve objectives (handouts/ slides, etc)	
Please indicate the overall effectiveness of this activity by indicating your agreement with the following statements. 5 being strongly agree and 1 being strongly disagree:	
The presentation and facilitated discussion enhanced my knowledge, skills and attitudes.	
I learned about and from other members of the Clinical Lead Forum.	
The Clinical Lead Forum met my personal expectations.	
I intend to make clinical practice change(s) as a result of information learned during this session.	
Attending the Clinical Lead Forum helped me feel an increased sense of community and belonging.	
Please assign an overall score of the session, 5 being excellent and 1 being poor.	
Please provide additional comments/constructive feedback for facilitators or organizers of CLF.	
Suggestions for future Clinical Lead Forum topics:	

Clinical Lead Forum Participant Assessment
COVID-19 Series

Thank you for participating in the Clinical Lead Forum. Please complete this optional survey to help us evaluate the educational program. If you fill out this anonymous survey, you are agreeing to take part in this educational research activity. Your decision to participate, or not participate, in the Forum and optional surveys will have no impact on your employment status or job performance evaluations. Thank you for your feedback.

Date of Clinical Lead Forum	
Topic of Clinical Lead Forum	
Facilitator(s)	
Professional Category/Degree	MBBS/ MBChB/ MD/ DO/- in practice<br>MBBS/ MBChB/ MD/ DO/- resident/fellow<br>Pharmacist<br>PhD/PsyD/EdD/DrPH<br>Medical student<br>Nurse<br>Other
Where are you currently based?	Argentina<br>Botswana<br>Colombia<br>Eswatini<br>Lesotho<br>Malawi<br>Romania<br>Tanzania – Mbeya<br>Tanzania - Mwanza<br>Uganda<br>USA<br>Other
What is your role in the COVID-19 response? Please choose the category that best applies.	Executive Director (ED)<br>ED-appointed Focal Person<br>Clinical Staff<br>Operations Staff<br>Subject Matter Expert<br>US-based Technical Support (including BCM/TCH)<br>Global Health Corps Physician<br>Global Health Resident<br>Other (please specifcy)
Did this presentation and facilitated discussion emphasize new concepts?	Yes<br>No<br>Unsure
Did this presentation and facilitated discussion refresh previous knowledge?	Yes<br>No<br>Unsure

Do you intend to make any clinical practice change(s) as a result of information learned during this session?	Yes<br>No<br>Unsure<br>Not applicable – non-clinical respondent
Did the facilitated discussion adequately address the limitations and opportunities inherent to the local health system?	Yes<br>No<br>Unsure
Did the facilitated discussion outline realistic strategies to improve clinical outcomes given the limitations and opportunities inherent to the local health system?	Yes<br>No<br>Unsure
Please assign an overall score of the presentation: 1 (needs major improvement) to 10 (exceptional)	1 2 3 4 5 6 7 8 9 10
Did attending the series make you feel an increased sense of community?	Yes<br>No
Did you learn new information or knowledge from the series?	Yes<br>No
If yes, how did you apply the information or knowledge in your practice?	[open-ended]
Have you started, been a part of, or know of a new initiative that has started as a result of knowledge sharing in the series?	Yes<br>No
If yes, please describe.	[open-ended]
Please rate the following statement:<br><br>Attending the COVID-19 series helped me stay informed about the developments in the field.<br><br>Attending the COVID-19 series helped me feel an increased sense of community.	Strongly agree<br>Somewhat agree<br>Neutral<br>Somewhat disagree<br>Strongly disagree
Please provide additional comments and constructive feedback for the facilitators and/or organizers of the session to improve future clinical lead forums.	
Suggestions for future Clinical Lead Forum topics (COVID-19 or non-COVID-19):	

Clinical Lead Forum Facilitator Assessment

Thank you for participating in the Clinical Lead Forum. Please complete this optional survey to help us evaluate the educational program. If you fill out this anonymous survey, you are agreeing to take part in this educational research activity. Your decision to participate, or not participate, in the Forum and optional surveys will have no impact on your employment status or job performance evaluations. Thank you for your feedback.

Date of Clinical Lead Forum	
Topic of Clinical Lead Forum	
Facilitator(s)	
Professional Category/Degree	MBBS/ MBChB/ MD/ DO/- in practice<br>MBBS/ MBChB/ MD/ DO/- resident/fellow<br>Pharmacist<br>PhD/PsyD/EdD/DrPH<br>Medical student<br>Nurse<br>Other
Foundation where you are based	Argentina<br>Botswana<br>Eswatini<br>Lesotho<br>Malawi<br>Romania<br>Tanzania – Mbeya<br>Tanzania - Mwanza<br>Uganda<br>BIPAI HQ<br>Other
Preparing for the presentation and facilitated discussion was a valuable use of my time.	Yes<br>No<br>Unsure
I received adequate mentorship and support to prepare for the presentation and facilitated discussion.	Yes<br>No<br>Unsure
I learned something new from the discussion between sites about my presentation.	Yes<br>No<br>Unsure
Presenting and facilitating discussion is valuable for my professional development.	Yes<br>No<br>Unsure
Please provide additional comments and constructive feedback for the organizers of the session to improve future Clinical Lead Forums.	
Suggestions for future Clinical Lead Forum topics:	
